# Integrated Tissue and Blood miRNA Expression Profiles Identify Novel Biomarkers for Accurate Non-Invasive Diagnosis of Breast Cancer: Preliminary Results and Future Clinical Implications

**DOI:** 10.3390/genes13111931

**Published:** 2022-10-24

**Authors:** Fei Su, Ziyu Gao, Yueyang Liu, Guiqin Zhou, Ying Cui, Chao Deng, Yuyu Liu, Yihao Zhang, Xiaoyan Ma, Yongxia Wang, Lili Guan, Yafang Zhang, Baoquan Liu

**Affiliations:** 1 College of Bioinformatics Science and Technology, Harbin Medical University, Harbin 150081, China; 2 Department of Anatomy, Harbin Medical University, Harbin 150081, China; 3 Department of Immunology, Harbin Medical University, Harbin 150081, China; 4 Department of Information Management, Shanghai Lixin University of Accounting and Finance, Shanghai 200000, China; 5 Department of Modern Medicine and Pharmacy, University of Tibetan Medicine, Lhasa 850000, China

**Keywords:** tissue, blood, breast cancer, primary synergy miRNA, diagnostic marker

## Abstract

We aimed to identify miRNAs that were closely related to breast cancer (BRCA). By integrating several methods including significance analysis of microarrays, fold change, Pearson’s correlation analysis, *t* test, and receiver operating characteristic analysis, we developed a decision-tree-based scoring algorithm, called Optimized Scoring Mechanism for Primary Synergy MicroRNAs (O-PSM). Five synergy miRNAs (hsa-miR-139-5p, hsa-miR-331-3p, hsa-miR-342-5p, hsa-miR-486-5p, and hsa-miR-654-3p) were identified using O-PSM, which were used to distinguish normal samples from pathological ones, and showed good results in blood data and in multiple sets of tissue data. These five miRNAs showed accurate categorization efficiency in BRCA typing and staging and had better categorization efficiency than experimentally verified miRNAs. In the Protein-Protein Interaction (PPI) network, the target genes of hsa-miR-342-5p have the most regulatory relationships, which regulate carcinogenesis proliferation and metastasis by regulating Glycosaminoglycan biosynthesis and the Rap1 signaling pathway. Moreover, hsa-miR-342-5p showed potential clinical application in survival analysis. We also used O-PSM to generate an R package uploaded on github (SuFei-lab/OPSM accessed on 22 October 2021). We believe that miRNAs included in O-PSM could have clinical implications for diagnosis, prognostic stratification and treatment of BRCA, proposing potential significant biomarkers that could be utilized to design personalized treatment plans in BRCA patients in the future.

## 1. Introduction

Breast cancer (BRCA) is the most common cancer worldwide [1,2,3,4,5], accounting for 11.7% of new cancer cases, which has risen rapidly [6,7]. To diagnose BRCA in its early stages, reduce its mortality, and reduce treatment-related harm to patients [8,9], a current challenge is to identify noninvasive and accurate biological markers to be used as indicators for early screening and diagnosis of BRCA. 

MicroRNAs (miRNAs) are a class of non-coding single-stranded RNA molecules with a length of approximately 22 nucleic acids [1,10,11,12], which can be found in tissues and blood [13,14,15]. For patients with different cancers, tumor-specific or related changes have been found in the free nucleic acids in blood circulation [16,17]. MiRNAs constitute short non-coding RNAs of post-transcriptional regulatory genes [18,19,20] and play an important role in development [21] and in normal physiological activities, and they can act as carcinogens [22,23] or tumor inhibitory regulators [24,25]. An increasing number of studies have recently reported close associations between miRNAs and cancers [25,26]. According to REMARK’s suggestion [27], we aimed to identify miRNAs closely related to BRCA as clinical diagnostic markers [1,28,29,30].

The majority of previous studies have focused on tissues [31,32] or blood [33,34] to search for cancer-related markers, but we hope to identify markers that have significantly combined properties in both tissues and blood, to increase diagnostic accuracy. Blood-derived markers [35,36,37,38,39] are not stable and may show opposite trends in different studies. Tissue-derived markers perform more consistently, but tissue sampling can be physically damaging to patients. Therefore, we hope to find markers of tissue and blood coexistence for BRCA identification and diagnosis. Furthermore, we searched for “tissue-blood shared miRNAs as cancer biomarkers” regarding BRCA or other carcinomas, but there were only a few studies that focus on marker miRNAs that work in both tissues and blood [40,41,42,43,44]. Thereby, we hope to fill the gap of miRNAs shared by tissues and blood as tumor biomarkers. 

In this study, we integrated many methods—Significance Analysis of Microarrays (SAM), fold change (FC), Pearson’s correlation analysis, *t* test, receiver operating characteristic (ROC), and decision trees—to develop a new method, called Optimized Scoring Mechanism for Primary Synergy MicroRNAs (O-PSM), that can identify disease-related miRNAs that a play major role in BRCA. This algorithm was performed for each feature selection set, first filtering key miRNAs using SAM, FC, Pearson’s correlation analysis, and *t* test, then constructing decision trees to obtain synergistic miRNAs, and in each tree, further selecting miRNA sets to ensure they lead to maximum purity at each branch of the nodes. This binary tree was split until it stopped growing; thus, the miRNAs in the tree represent a set of feature combinations from the root node to the leaf nodes that contribute synergistically to the classification. The combined miRNAs classify the samples sequentially according to the hierarchical structure of the tree and jointly decide to identify the diagnosis of BRCA. 

We used O-PSM to process and analyze BRCA data obtained from The Cancer Genome Atlas (TCGA) and tested the classification performance in tissues and blood using data obtained from the Gene Expression Omnibus (GEO), to identify five BRCA-related miRNAs: hsa-miR-139-5p, hsa-miR-331-3p, hsa-miR-342-5p, hsa-miR-486-5p, and hsa-miR-654-3p, which exhibited satisfactory classification efficiencies in both tissues and blood. The highest single area under the curve (AUC) of these five miRNAs was the AUC of hsa-miR-139-5p, which was equal to 0.9941 with a CI of 98.44–100%, and combined AUC of 0.9975 with a CI of 99.36–100%. In independent sets, the highest single AUC was 0.8793 (CI: 78.69–97.16%) from hsa-miR-486-5p, and the combined AUC was 0.9105 (CI: 99.36–100%) in serum, with 0.9714 (CI: 93.4–100%) from hsa-miR-139-5p and 1.0000 (CI: 99.36~100%) in the tissue independent set 1, 0.9714 (CI: 93.4–100%), from hsa-miR-139-5p and 1.0000 (CI: 99.36–100%) in tissue independent set 2.

We also assessed the categorization efficiency of the five screened miRNAs for BRCA staging and typing, the latter being the one better characterized, surprisingly, by the panel. In addition, we compared hsa-miR-125a-5p [45,46] and hsa-miR-146b-5p [45,47], which were experimentally confirmed in tissues and blood from previous reports. The results showed that miRNAs identified by O-PSM had better categorization efficiency. We also analyzed the target genes of five miRNAs and obtained Protein-Protein Interaction (PPI) networks and functional enrichment of target genes by STRING and Metascape databases. In addition, survival analysis showed that high expression of hsa-miR-342-5p had a great impact on patient prognosis. Our results showed that the combined use of the panel of identified miRNAs may be effective for non-invasive diagnosis and prognostic stratification of patients, in order to design a novel biomarkers-based approach for BRCA management in clinical practice. 

## 2. Materials and Methods

### 2.1. Data

**Figure 1:**
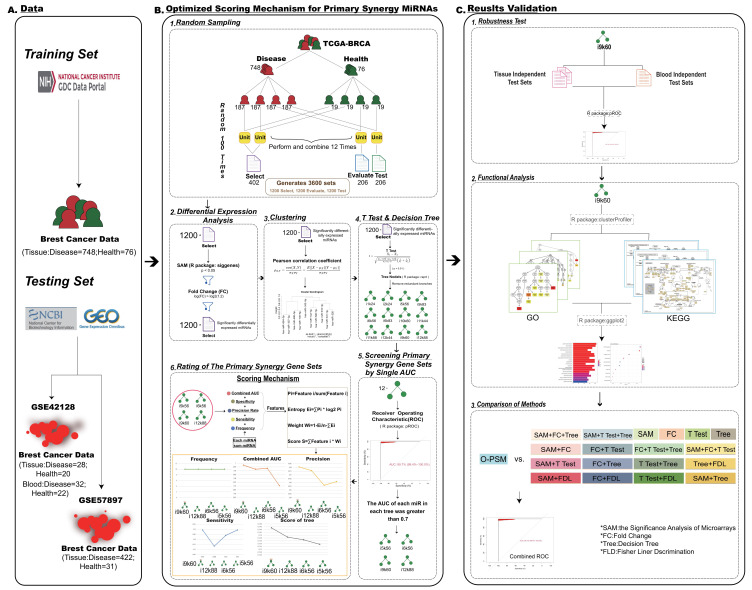
Flow Chart. (**A**) Data acquisition. (**B**) The set of microRNAs (miRNAs) with major synergistic effect was screened by the synthesis algorithm Optimized Scoring Mechanism for Primary Synergy MicroRNAs (O-PSM). 1. Four-fold cross sampling. 2. Differential expression analysis. 3. Clustering was performed using the pearson correlation coefficient. 4. The significant decision tree models were obtained by *t* test and decision tree. 5. Screening primary synergy gene sets by single Area Under Curve (AUC) of each miRNA > 0.7. 6. After rating the primary synergy gene sets, keep the highest scoring marker set. (**C**) Results validation.

The TCGA BRCA RNA-seq data were obtained from the UCSC Xena database (https://xenabrowser.net/ (accessed on 1 October 2020)), which included data from 748 disease patients, 187 healthy patients, and a total of 2253 miRNAs. The prognostic data and phenotype information were also downloaded from the UCSC Xena database. There were three independent test sets in the GEO (https://www.ncbi.nlm.nih.gov/geo/ (accessed on 1 October 2020)) for the classification effectiveness test; two of them involved tissue data, and one involved blood data. The tissue independent set 1 was from GSE42128 [48] platform 3 (GPL15018 Agilent-031181 Unrestricted_Human_miRNA_V16.0_Microarray 030840), with 1205 miRNAs, which had data from 28 cancer patients and 20 para-carcinoma patients. The tissue independent set 2 was from GSE57897 [49] (GPL18722→Homo sapiens microRNA array), with 1849 miRNAs, which had data from 422 cancer patients and 31 healthy controls. The blood data were from GSE42128 platform 2 (GPL16224→Exiqon LNA RT-PCR Human panels (1 and 2)), with 274 miRNAs, which had preoperative serum data from 32 cancer patients and 22 healthy controls (Figure 1A).

We used GSE81002 [50,51,52] (GPL10656 Agilent-029297 Human miRNA Microarray v14 Rev.2 (miRNA ID version)) and GSE97811 [53] (GPL21263 3D-Gene Human miRNA V21_1.0.0, microarray data) from GEO to validate the classification effect of the screened miRNAs for BRCA typing and staging. GSE81002 (GPL10656), which has 128 miRNAs overlapping with TCGA BRCA data, and all 5 marker miRNAs in it, left a total of 425 samples. We removed 50 samples without pam50 subtype; then, there were 45 basal-like samples, 44 normal-like samples, 155 Luminal A samples, 89 Luminal B samples, and 42 HER-2 (+) samples, 375 samples in total. GSE97811, which has 2222 miRNAs overlap with TCGA BRCA data, and all 5 marker miRNAs in it, consisted of 61 samples, including 28 stage 1 samples, 28 stage 2 samples, and 5 stage 3 samples. 

### 2.2. Random Sampling

Random sampling was performed using BRCA data downloaded from TCGA. The diseased and healthy data were divided into four parts for random combination; three were training sets and one was a test set. To enrich our research samples and generate more accurate results, two-thirds of the training set was divided into a feature selection set, and one-third was the feature evaluation set. One sampling produced 12 feature selection sets, and we sampled 100 times at random to produce 1200 feature selection sets. We finally obtained 1200 feature selection sets (i1k1 − i12k100), 1200 feature evaluation sets (i1k1 − i12k100), and 1200 internal test sets (i1k1 − i12k100), to use in subsequent data analyses (Figure 1B). I is the number of permutations of random combinations, k is the number of random samples. 

### 2.3. Differential Expression Analyses

The SAM method (R Package: siggenes) was then used to select differentially expressed miRNAs from 1200 features selection sets, and characteristic miRNAs with *p* < 0.05 were identified in each set. FC was performed on the characteristic miRNAs obtained from SAM in each set, and the threshold was set as log_2_(|FC value|) > log_2_(1.2), to identify the miRNAs with significant differential expressions (Figure 1B). 

### 2.4. Clustering

To further obtain co-expression relationships between the miRNAs identified by SAM and FC, these miRNAs were clustered using Pearson’s correlation analysis with correlation coefficient (r) > 0.6, to obtain the sets of co-expressed characteristic miRNAs (Figure 1B). 

### 2.5. The T Test and Decision Tree

The unpaired *t* test (*p* < 0.01) was performed on the clustering results, and the characteristic miRNAs from each set identified by this test were used for the decision trees (R package: rpart). To identify more significant sets, we also removed trees with redundant branches (Figure 1B). 

### 2.6. Screening Primary Synergy miRNA Sets by Single AUCs

We used ROC analyses to select miRNA sets. The AUC of the trees were calculated using the R package, pROC. AUC value, accuracy and sensitivity were obtained from each set. To select more representative primary synergy miRNAs, the single AUC value of each miRNA in the feature evaluation set was greater than 0.7 during standard screening (Figure 1B). 

### 2.7. Ratings of the Primary Synergy miRNA Sets

The combined AUC value, specificity, accuracy, and sensitivity of each miRNA in the remaining feature evaluation sets and the frequency of occurrence of each miRNA was combined to score the sets. The entropy weighting method was used to transform these indices into scores of the primary synergy miRNA sets (Figure 1B).

(1) $$P_{xy}=m_{xy}\;/\;\left(\sum_{x}m_{xy}\right)$$

*x* is for each feature set, *y* is the indices, *y* ∈ (1, 2, 3, …, n). $m_{xy}$ is set as the AUC value, specificity, accuracy, sensitivity and frequency of miRNA in the feature set.

(2) $$E_{y}=-\sum_{x}P_{xy}\cdot \;log\left(P_{xy}\right)$$

$E_{x}$ is the entropy.

(3) $$W_{y}=\left(1-E_{y}\right)\;/\;\left(5-\sum \;E_{y}\right)$$

$W_{x}$ is the weight of entropy. Then, we assigned entropy weight to each set for subsequent scoring.

(4) $$S_{x}=\sum_{y}m_{xy}\cdot \;W_{y}$$

The scores ($S_{x}$) of each miRNA set were calculated and compared. The set with the highest score was identified as the one with significant synergistic effects. 

We selected the decision tree method, not only because it showed the synergistic effect of miRNAs, but also because it provided us with the primary and secondary relationships between them. The root node was the node with the most important significance in the whole tree. 

### 2.8. O-PSM for Primary Synergy miRNAs

By integrating the series of methods including random sampling, SAM, FC, Pearson’s correlation analysis, *t* test, ROC and decision tree, we proposed the decision tree-based scoring algorithm, which involves the aforementioned steps (Figure 1A,B). 

### 2.9. Comparison of Methods

We compared O-PSM with a range of methods, including SAM, FC, *t* test, decision tree and their combinations, for a total of 19 methods, including the use of Fisher Liner Discrimination and random forest to further determine the advantages of O-PSM (Supplementary Table S7). Additionally, we also evaluated the robustness of O-PSM by randomly permutating the data class labels. When the number of miRNAs screened by the compared methods exceeded 5, we randomly selected 5 miRNAs for comparison.

### 2.10. Functional Annotation of miRNAs

We obtained a total of 2752 target genes for 5 miRNAs from the miRTarBase database (https://mirtarbase.cuhk.edu.cn/~miRTarBase/miRTarBase_2022/php/index.php (accessed on 26 March 2021)). Target genes that were significantly negatively correlated with markers were screened by Pearson’s correlation coefficient. Using the negatively correlated target genes, we enriched Gene Ontology (GO) terms and Kyoto Encyclopedia of Genes and Genomes (KEGG) pathways for differentially high expressed miRNAs (hsa-miR-342-5p and hsa-miR-331-3p) and differentially low expressed miRNAs (hsa-miR-139-5p, hsa-miR-486-3p and hsa-miR-654-3p) using the R package “clusterProfiler”, respectively, to obtain relevant functional annotations. 

### 2.11. Construction of PPI Network and In-Depth Network Analysis

We obtained the target genes of these 5 miRNAs by querying miRTarBase (https://mirtarbase.cuhk.edu.cn/~miRTarBase/miRTarBase_2022/php/index.php (accessed on 26 March 2021)), with 354 of them in the STRING database (https://string-db.org (accessed on 28 March 2021)). We used these 354 target genes to build a PPI network and analyze their mechanisms. In order to obtain more information and make the PPI network clearer, we utilized Cytoscape software to visualize it.

Molecular Complex Detection (MCODE) in Cytoscape was used to explore hub modules of PPI network, with the threshold degree cut-off  =  2, node score cut-off  =  0.2, k-core  =  2, and max depth  =  100. 

Finally, we obtained the functional annotation and the pathway enrichment of those target genes in Metascape (https://metascape.org (accessed on 28 March 2021)). 

### 2.12. Survival Analysis

Using TCGA BRCA prognostic information, of which there were 741 samples, we first analyzed the effect of different clinical characteristics on patient survival by univariate cox regression. Then, we collected factors that were associated statistically significantly with the survival status of BRCA patients. We then combined remarkable factors in a multivariate cox regression analysis to obtain independent factors that could influent the prognosis of patients. Analysis was performed using the “survival” package and “survminer” package in R. Then, we plotted Kaplan-Meier (K-M) curve by R package “ggsurvplot”.

## 3. Results

### 3.1. Identification of miRNAs with Primary Synergy Using O-PSM of Tissues

**Figure 2:**
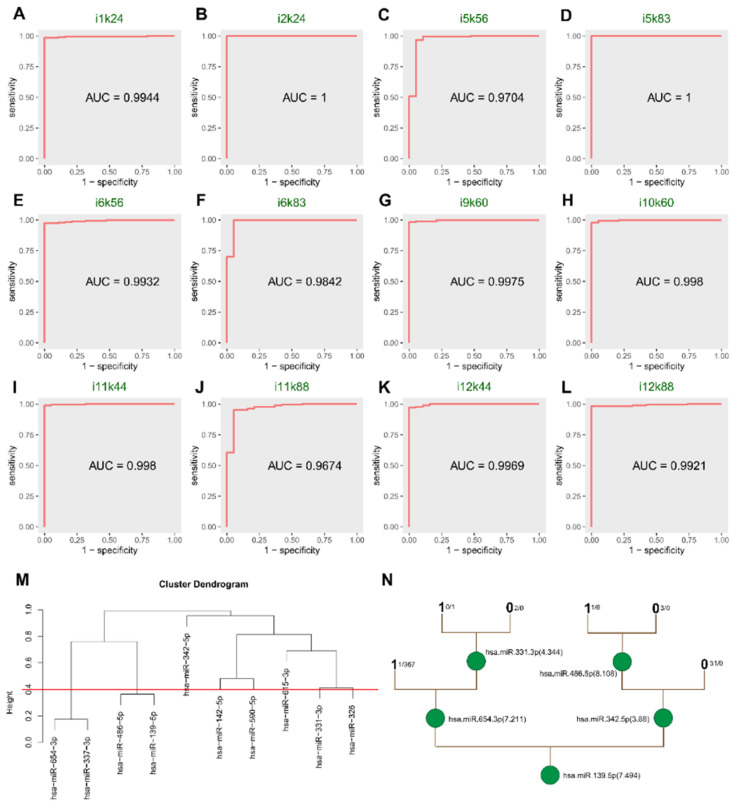
ROC of 12 trees in evaluated sets and cluster and tree plots of i9k60. (**A**–**L**) The ROC of 12 decision trees without redundant branches in the evaluated sets. (**M**) The 10 differentially expressed in i9k60 miRNAs were divided into 8 co-expression sets by Pearson’s correlation coefficient analysis. (**N**) Unpaired *t* test (*p* < 0.01) was performed on the clustering results, the characteristic miRNAs in i9k60 screened by *t* test were used for the decision tree (R package: rpart). Through pruning the tree, we ended up with 5 miRNAs: hsa-miR-139-5p, hsa-miR-331-3p, hsa-miR-342-5p, hsa-miR-486-5p, and hsa-miR-654-3p.

By performing O-PSM, 1200 sets were obtained, trees were generated in each feature selection set, and trees with redundant branches were subsequently removed. To determine the efficiency of discrimination, ROCs in the feature evaluation sets were calculated for the remaining 12 miRNA sets without redundant branches of the trees involving i1k24, i2k24, i5k56, i5k83, i6k56, i6k83, i9k60, i10k60, i11k44, i11k88, i12k44, and i12k88 (Figure 2A–L, Supplementary Table S1). 

To identify the miRNA sets with high quality, the sets with each single AUC value greater than 0.7 were identified. The combined ROC, specifically, accuracy of each miRNA set, and frequency of occurrence of each miRNA in all miRNA sets were used to score using O-PSM. Finally, the highest ranked set was identified as i9k60, with a score of 0.7452. 

There were 41 miRNAs in the raw data of i9k60. SAM selected 30 miRNAs with *p* < 0.05. After FC screening (|FC value| > 1.2), 10 miRNAs remained. Using clustering, eight co-expression sets were identified. All sets had *t* test *p* values less than 0.01. After pruning the decision tree, we had five miRNAs (hsa-miR-139-5p, hsa-miR-331-3p, hsa-miR-342-5p, hsa-miR-486-5p, and hsa-miR-654-3p) that had major synergistic effects on BRCA. The hsa-miR-139-5p was the miRNA that had the highest single AUC (0.9941), and it was also the root node of the tree, which confirmed the importance of hsa-miR-139-5p. 

### 3.2. Significant Categorization of Tissues

**Figure 3:**
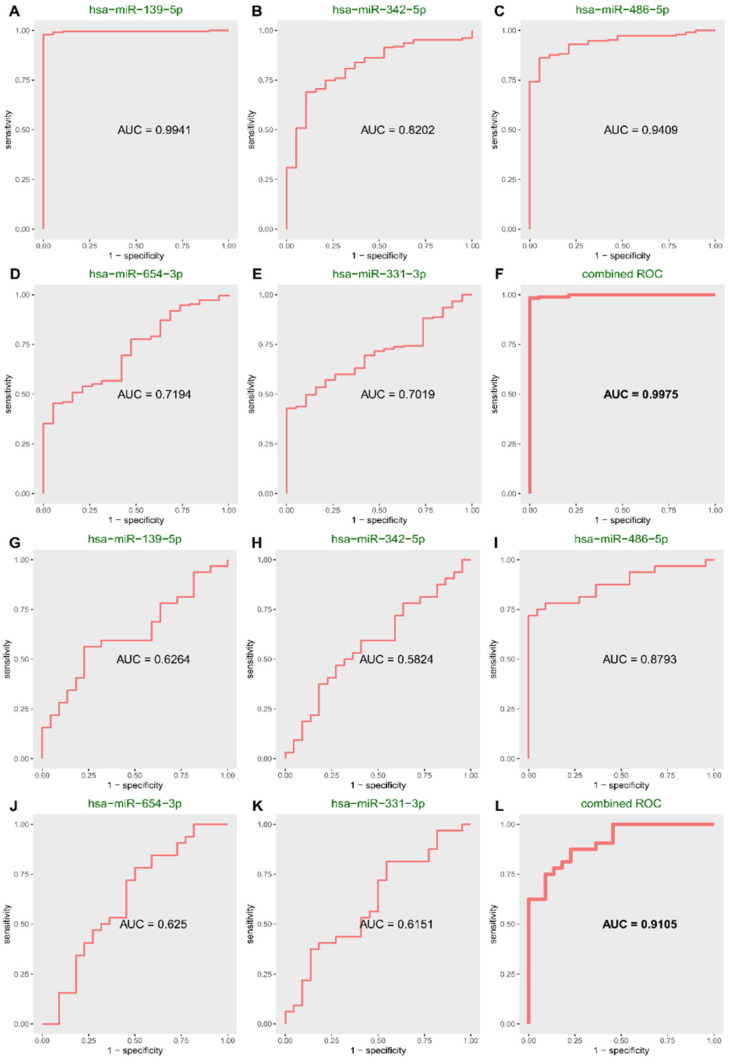
The single ROC of each miRNA and their combined ROC in the evaluated set and blood independent test set of i9k60. (**A**–**E**) Single ROC of each miRNA in evaluated set of i9k60. (**F**) Combined ROC of the 5 miRNAs in evaluated set of i9k60. (**G**–**K**) Single ROC of each miRNA of i9k60 in blood independent test set. (**L**) Combined ROC of the 5 miRNAs of i9k60 in blood independent test set.

In the internal evaluated sets, the combined AUC of those miRNAs reached 0.9975, exceeding the AUC of five single miRNAs, which showed that the combined effect was better than the individual effects (Supplementary Table S2 and Figure 3A–F). Hsa-miR-139-5p with the highest frequency was also the miRNA with the highest individual AUC. It was also the root node of the decision tree (Figure 2N), highlighting its significance among the five miRNAs. 

In the internal test sets, the results were the same as in the internal test set. The single AUC of hsa-miR-139-5p, hsa-miR-331-3p, hsa-miR-342-5p, hsa-miR-486-5p, and hsa-miR-654-3p are 0.9904, 0.7084, 0.7974, 0.9294 and 0.8224, respectively, and the combined AUC is 0.9930 (Supplementary Table S3 and Supplementary Figure S1). 

In independent validation set 1, the results were verified once (Supplementary Table S4 and Supplementary Figure S2), indicating the accurate classification performance of the miRNAs. The single AUC of hsa-miR-139-5p, hsa-miR-331-3p, hsa-miR-342-5p, hsa-miR-486-5p, and hsa-miR-654-3p are 0.9714, 0.5625, 0.6446, 0.8982 and 0.9125, the combined AUC is 1.0000.

To further test the combined effects of these five miRNAs, we tested them in tissue independent set 2. The AUC results of hsa-miR-139-5p, hsa-miR-331-3p, hsa-miR-342-5p, hsa-miR-486-5p, and hsa-miR-654-3p in GSE57897 showed that the combined effect reached 0.7181, exceeding each single AUC value, which showed the primary synergy of these five miRNAs again (Supplementary Table S5 and Supplementary Figure S3). 

### 3.3. Significant Categorization of Serum

The five miRNAs that showed primary synergy also achieved satisfactory results in the independent test sets of serum (GSE42128 platform 2), with 0.9105 for the combined AUC, indicating that the joint use of five miRNAs also had significant classification performance in serum (Supplementary Table S6 and Figure 3G–L). 

Both in tissues and blood (serum), the individual ROC was not as high as the combined value, indicating that the combined effect of these miRNAs was better than that of individual miRNAs. The results showed the reliable efficiency of O-PSM and synergy of these five miRNAs. 

### 3.4. Categorization Performed Remarkably Well in Typing and Staging of BRCA

For BRCA typing, GSE81002 was used to remove samples without subtype information. When samples of the subtype basal-like were cases, other subtypes were classified as controls. The same was true for other subtypes. The i9K60 miRNAs collection showed accurate categorization efficiency in basal-like BRCA and normal-like BRCA (Table 1). 

Next, we evaluated the classification efficacy of i9k60 miRNAs for different stages of BRCA. Similar to typing, when stage 1 BRCA samples were used as case group, samples at other stages were used as control. The same was performed for other stages. The analysis revealed that i9k60 miRNAs had better classification efficacy when in stage 2. The effect was not significant in other stages, and we speculated that this is due to the small amount of data (Table 2). 

### 3.5. Comparison with miRNAs That Had Been Experimentally Confirmed

**Figure 4:**
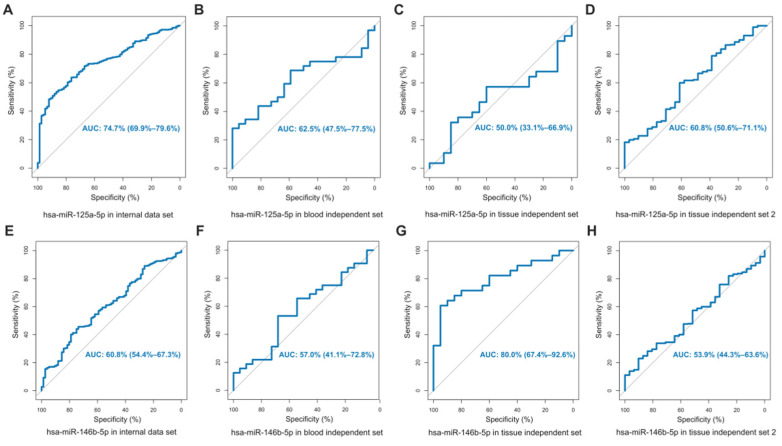
ROC of hsa-miR-125-5p and hsa-miR-146b-5p. (**A**–**D**) AUC for hsa-miR-125-5p was detected in TCGA-BRCA, blood independent set (GSE42128 platform2), tissue independent set 1 (GSE42128 platform3) and tissue independent set 2 (GSE57897). (**E**–**H**) AUC for hsa-miR-146b-5p was detected in TCGA-BRCA, blood independent set (GSE42128 platform2), tissue independent set 1 (GSE42128 platform3) and tissue independent Set 2 (GSE57897).

We compared the selected miRNA collections with those that had been experimentally verified to be closely related to BRCA, to further determine the collection abilities of i9k60 miRNAs. The miRNAs that met the requirements were in previous reports, showing that hsa-miR-125a-5p and hsa-miR-146b-5p simultaneously existed in tissues and blood. ROC was used to compare our sets of data with these previously reported data. The results showed that the categorization efficiency of hsa-miR-125a-5p and hsa-miR-146b-5p differed greatly from the miRNAs screened by us (Figure 4). For the miRNA set screened by O-PSM, not only was the AUC value of these five miRNAs alone better than hsa-miR-125a-5p and hsa-miR-146b-5p, but also the combined ROCs of the internal data set and the three independent validation sets were 0.9975, 1.0000, 0.7181, and 0.9105, respectively, which were all higher than hsa-miR-125a-5p and hsa-miR-146b-5p. The novel marker miRNAs we screened not only achieved good classification results in several data sets, but they also obtained great results when compared with the two experimentally validated BRCA-associated miRNAs. Therefore, we believed that the miRNAs screened by the O-PSM method are promising as markers for BRCA identification and diagnosis. 

### 3.6. Comparison of Methods

To test whether O-PSM had advantages over other methods, we compared it with a series of methods, including SAM, FC, *t* test, decision tree, and their combinations, 18 methods in total, including using Fisher Liner Discrimination to further identify the advantages of O-PSM (Supplementary Table S7). When the numbers of miRNAs screened by the comparison methods were more than five, we randomly selected five miRNAs for comparison. All the comparison results showed that there was a difference between these methods and O-PSM. The characteristic miRNAs screened by O-PSM had the highest combined AUC in TCGA-BRCA (0.9975), and the combined AUC in tissue independent validation set 1 tied with the AUC obtained by other partial methods and achieved better results in both tissue independent validation set 2 and blood independent validation set. Together, O-PSM showed good classification performance and could identify disease-related miRNAs with primary synergies. We also permutated the TCGA BRCA data to more accurately confirm the efficacy of these five miRNAs as clinical diagnostic markers for BRCA. However, the permutated data could not pass O-PSM, further validating the stringent screening conditions of O-PSM.

### 3.7. Functional Annotation

**Figure 5:**
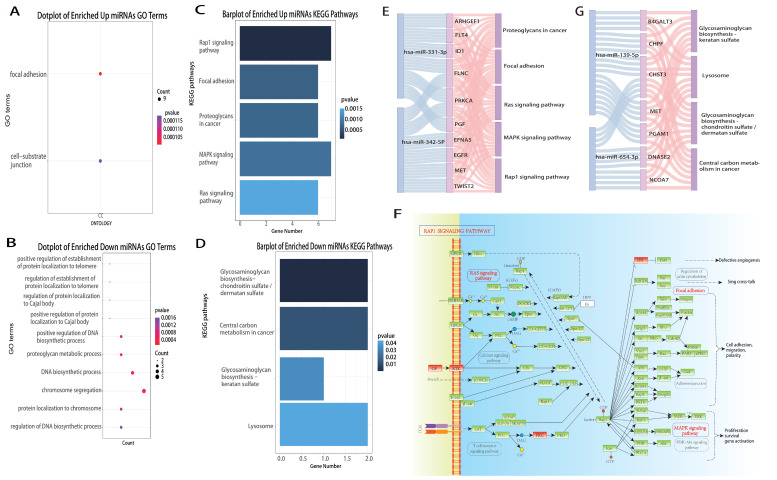
Gene Ontology (GO) and Kyoto Encyclopedia of Genes and Genomes (KEGG) annotations of hsa-miR-139-5p, hsa-miR-331-3p, hsa-miR-342-5p, hsa-miR-486-5p, hsa-miR-654-3p. (**A**,**B**) Through R package “clusterProfiler”, we obtained the GO annotation of these miRNAs. (**C**,**D**) Through R package “clusterProfiler”, we obtained the KEGG enrichment analysis of them. (**E**) Sankey plot of upregulated miRNAs—target genes—pathways. (**F**) KEGG pathways plot of target genes of upregulated miRNAs. (**G**) Sankey plot of downregulated—target genes—pathways.

The development, progression and metastasis are highly complex processes that involve multiple biological functions and pathways. We queried the target genes of marker miRNAs in miRTarBase, screened the target genes negatively co-expressed with markers by Pearson’s correlation coefficient, and annotated miRNAs functionally according to the significant negatively co-expressed target genes. Then, we performed GO and KEGG functional annotation by R package “clusterProfiler” for differentially high expressed miRNAs (hsa-miR-342-5p and hsa-miR-331-3p) and differentially low expressed miRNAs (hsa-miR-139-5p, hsa-miR-486-3p and hsa-miR-654-3p). We found that four out of five marker miRNAs participate in multiple biological procedures and pathways that may affect BRCA progression by regulating target genes (Figure 5).

Based on GO (Figure 5A,B) and KEGG (Figure 5C,D), we identified the upregulated miRNAs involved in the Rap1 signaling pathway, focal adhesion, Ras signaling pathway, MAPK signaling pathway and proteoglycans in cancer (Figure 5E), which were closely related to cell adhesion and other related functions. Adhesion is associated with cancer metastasis [54]. Cell migration is central to numerous physiological processes, including embryonic development, immune surveillance and wound healing, and dysregulation of migration is critical for cancer propagation. Among the target genes, *EGFR* is epidermal growth factor receptor [55,56,57], which is frequently expressed at high levels in different forms of cancer, and its expression is often positively correlated with cancer progression and poor prognosis [58,59,60,61,62,63]. *FLT4* is FMS-like tyrosine kinase 4 [64], *ID1* is a DNA-binding protein inhibitor [65], *MET* is proto-oncogene tyrosine protein kinase Met [66], and *PRKCA* is the classical protein kinase C alpha type [67,68,69], which are receptor tyrosine kinases (RTKs). RTKs are cell surface receptors with specific structural and biological characteristics that react to environmental clues by activating proper signaling cascades in cancer cells. *PGF* is placental growth factor [70], which belongs to growth factors (GF). In cancer cells, *PGF* mediates a range of pro-metastatic cellular events, including engaging endothelial cells to build blood supply, enhancing invasiveness and cell movement [71]. The upregulated markers reduce the expression of target genes, leading to dysregulation of processes such as signal transduction, cellular processes, and human diseases, and inhibit functions such as cell adhesion, which may promote tumor metastasis (Figure 5F).

The target genes of downregulated miRNAs are mainly involved in glycosaminoglycan biosynthesis [72,73] and lysosome [74] and central carbon metabolism in cancer [75]; and these functions are tightly related to angiogenesis, cell growth, cell proliferation and apoptosis (Figure 5G). We assumed that marker miRNAs downregulate expression, reduce the repressive effect on target genes, enable the overactivation of related pathways and indirectly promote tumor development. 

### 3.8. PPI Network and Mechanism Analysis of Target Genes

**Figure 6:**
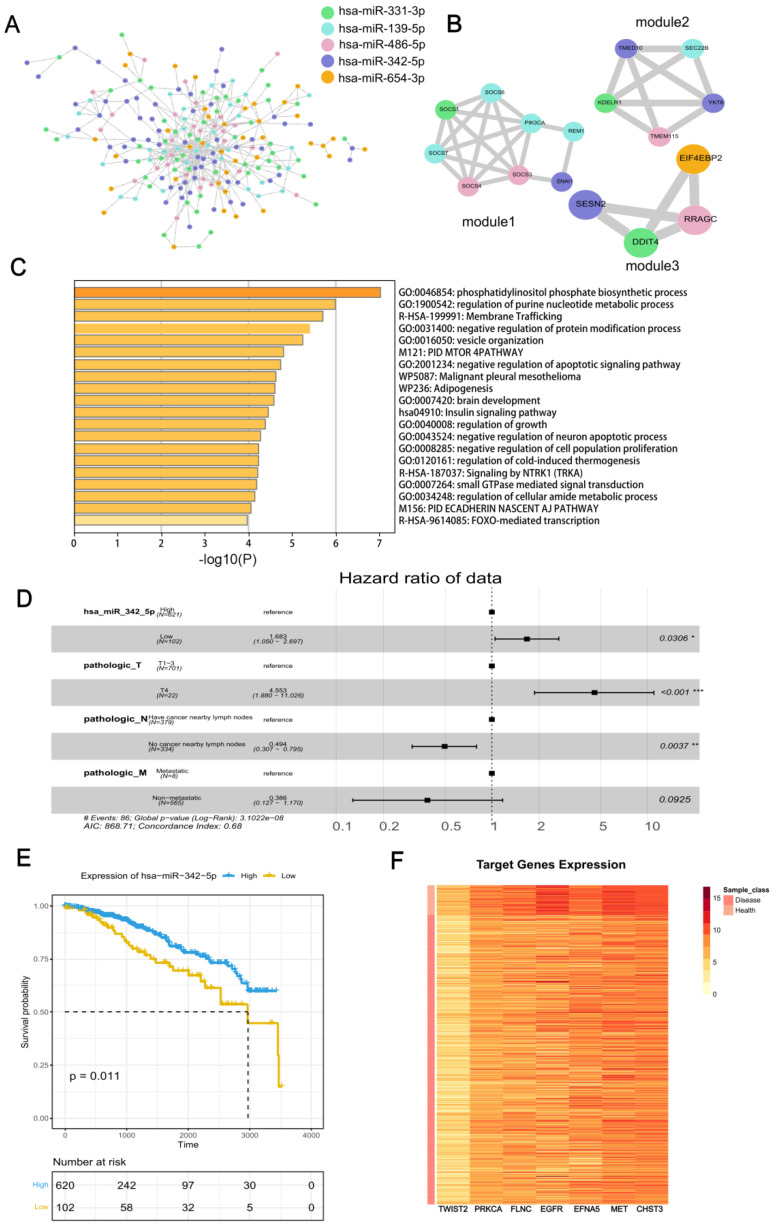
Protein-Protein Interaction (PPI) network and mechanism of the target genes and survival analysis of miRNAs. (**A**) PPI network of target genes obtained from STRING database. (**B**) The top three clusters filtered by MCODE. (**C**) Functional annotation of target genes by Metascape. (**D**) Multivariate cox regression analysis of factors that influence prognosis of BRCA patients. ‘*’: *p* < 0.05, ‘**’: *p* < 0.01, ‘***’: *p* < 0.001. (**E**) Prognostic impact of high and low expression of hsa-miR-342-5p on TCGA BRCA patients. (**F**) Heat map of significantly differentially expressed hsa-miR-342-5p target genes in BRCA patients and normal samples.

With the STRING database, we obtained the PPI network of target genes (Figure 6A), in the whole network, among them, the target genes that possessed the most interactions are targets of hsa-miR-342-5p, which have 65 relationships. The first three tightly connected hub interworking subnets are filtered by MCODE (Figure 6B–D). 

Then, we obtained the functional annotations of all target genes of five marker miRNAs in Metascape, and the results (Figure 6E) showed that they are highly related to the phosphatidylinositol phosphate biosynthetic process with the *p* value of 9.886 × 10^−8^. Phosphatidylinositol 3-phosphate is a lipid that regulates membrane dynamics protein sorting and cell signaling [76]. They are also related to regulation of the purine nucleotide metabolic process (*p* = 1.028 × 10^−6^). Purines are basic components of nucleotides in cell proliferation; thus, impaired purine metabolism is associated with the progression of cancer, membrane trafficking (*p* = 1.999 × 10^−6^), which is a focal point for targeting cancer [77], negative regulation of the protein modification process (*p* = 3.963 × 10^−6^), vesicle organization (*p* = 5.970 × 10^−6^), PID MTOR 4PATHWAY (*p* = 1.629 × 10^−5^), negative regulation of apoptotic signaling pathway (*p* = 1.897 × 10^−5^), malignant pleural mesothelioma (*p* = 2.421 × 10^−5^), adipogenesis (*p* = 2.489 × 10^−5^) and brain development (*p* = 2.630 × 10^−5^).

Through the PPI network mining modules, we found that the first three modules all contain the target genes of hsa-miR-342-5p; thus, we think that the differential expression of hsa-miR-342-5p may have a more important effect on BRCA. Therefore, we analyzed the effect of hsa-miR-342-5p on the prognosis of BRCA patients.

First, we determined the factors that would affect the prognosis of BRCA patients by univariate cox regression. The analysis revealed that hsa-miR-342-5p as well as primary tumor stage (T stage), regional lymph nodes stage (N stage) and distant metastasis (M stage), which are also known as the TNM stage, were associated statistically significantly with the survival status of BRCA patients (Figure 6D). The effect of gender, race and radiation therapy on patient prognosis was not remarkable. We then combined hsa-miR-342-5p with the TNM stage in a multivariate cox regression analysis, and the results showed that hsa-miR-342-5p remarkably influenced patient survival despite the influence of TNM stage (Figure 6D). Thus, we concluded that hsa-miR-342-5p could influence the prognosis of BRCA patients significantly as an independent factor. By plotting the K-M curve, we could see that when hsa-miR-342-5p was highly expressed in patients, there was better prognosis of patients (Figure 6E).

We then analyzed the expression of target genes of hsa-miR-342-5p, which significantly enriches pathways, and could see that their target genes were remarkably low expressed in disease and relatively high expressed in normal samples, consistent with our findings from functional and survival analyses (Figure 6F).

## 4. Discussion

Identifying non-invasive markers of tumors has been a pressing issue. In urologic tumors, miRNAs have been shown to be useful biomarkers, but a large body of research data is not yet available for clinical practice [78]. However, it is now clear to us that different cancers can have very different clinical presentations in different patients. The use of biomarkers can be a very promising strategy. Epigenetics-based biomarkers such as dysregulated DNA methylations, deregulated expression of chromatin structure proteins and miRNAs or nt-RNAs or lncRNAs could have a high impact on clinical practice in oncology. Nevertheless, the transfer from laboratory to clinical practice remains slow. This is why translational research, with clinical implications, is the future of oncology research.

Avan et al. had compiled the potential value of tissue and circulating miRNAs for prognostic and therapeutic applications in BRCA in 2018 [79], and they concluded that miRNAs are promising for early detection of BRCA, predicting prognosis, and monitoring patient response to treatment based on preclinical and clinical investigations of tissue-specific miRNAs and circulating miRNAs. However, the performance of circulating miRNAs has been inconsistent and may lead to conflicting conclusions in different studies. Tissue-specific miRNAs are relatively stable, but biopsy can cause some damage to the body. We hope to mine BRCA markers in miRNAs common to tissue and blood, so that marker miRNAs can have the advantages of both: non-invasive and stable.

We proposed O-PSM to identify more efficient and accurate non-invasive disease-related miRNAs. Five miRNAs (hsa-miR-139-5p, hsa-miR-331-3p, hsa-miR-342-5p, hsa-miR-486-5p, and hsa-miR-654-3p) were identified from BRCA tissues and blood samples of patients, which played a major role in this disease. These five miRNAs showed precise classification performance in BRCA tissues and serum, showed appropriate significance for the clinical diagnosis of BRCA, and strongly supported the screening ability of O-PSM. We placed the entire O-PSM process into an R package stored at https://github.com/SuFei-lab/OPSM.git (accessed on 22 October 2021). Verification in tissues and blood confirmed that the combined effect of these five miRNAs provided a novel biomarker system for diagnosis of BRCA. Furthermore, the study suggested the possibility of using non-invasive methodologies to achieve an accurate diagnostic and prognostic definition of BRCA cases.

We verified the categorization efficacy of screened miRNAs in terms of BRCA typing [50,51,52] and staging [53], indicating that the collection of miRNAs with major synergistic effects obtained by O-PSM was closely related to BRCA. We also compared with experimentally verified miRNAs (hsa-miR-125a-5p [45,46] and hsa-miR-146b-5p [45,47]) related to BRCA. By comparing the miRNAs screened by O-PSM with those reported in the literature, it was determined that miRNAs obtained by O-PSM had better categorization efficiencies. Thus, we concluded that the fresh markers screened by O-PSM has good robustness.

We conducted preliminary survival analysis of hsa-miR-342-5p, which showed that the marker is statistically significantly related with prognosis of BRCA patients (*p* < 0.05), and the higher the marker expression, the better the survival outcome.

In addition to bioinformatic analysis, we also searched the literature in the hope of finding the relationship between marker miRNAs and anti-cancer drugs and exploring the mechanisms by which markers act as potential drug targets to influence patient treatment outcomes and prognosis. The search revealed that hsa-miR-342-5p regulates the expression of genes involved in tamoxifen-mediated apoptosis and cell cycle progression in tumor cells. Restoration of hsa-miR-342-5p expression may represent a novel therapeutic approach to sensitize and inhibit the growth of tamoxifen-refractory breast tumors [80]. Aside from BRCA therapeutics, we also retrieved marker miRNAs that play a role in other cancer treatments. Deng et al. found that hsa-miR-342-5p may act as a tumor suppressor in osteosarcoma (OS) by targeting *Wnt7b* to inhibit the effects on OS cells viability, migration, invasion, sensitivity to Doxorubicin and apoptosis [81]. Tang et al. found that hsa-miR-139-5p increased apoptosis and inhibited cisplatin (DDP), induced non-small cell lung cancer (NSCLC) cell proliferation in vitro by regulating the PI3K/AKT/caspase-3 signaling pathway, and sensitized NSCLC cells to DDP by targeting *HOXB2*. Modulation of hsa-miR-139-5p expression reversed DDP resistance and increased chemosensitivity of therapeutic NSCLC [82]. Furthermore, Fentanyl can inhibit the viability and invasion of NSCLC cells by inducing hsa-miR-331-3p and reducing *HDAC5* [83]. Hsa-miR-486-3p is an important mediator in regulating sorafenib resistance by targeting *FGFR4* and *EGFR*, thus providing a potential target for HCC treatment [84]. Allicin upregulates hsa-miR-486-3p and enhances the sensitivity of TMZ in glioblastoma. Allicin may be used as adjuvant chemotherapy for TMZ to improve patient prognosis, while hsa-miR-486-3p may be a potential target for glioblastoma treatment to improve outcomes [85]. Hsa-miR-654-3p enhances DDP sensitivity in Ovarian Cancer (OVC) cells by downregulating *QPRT* expression, and inhibition of hsa-miR-654-3p reverses the inhibitory effect of *QPRT*-targeted short interfering RNA on OVC cell proliferation and chemoresistance [86]. This shows that marker miRNAs participate in the treatment of diverse cancers, further demonstrating that our screened miRNAs may serve as potential drug targets for cancer treatment. We speculate that the drugs mentioned in our review may also have some implications for BRCA treatment.

Our study has a number of limitations: first, because TCGA BRCA data and GEO data (GSE42128) had little in common, it might have had an impact on the results. Nonetheless, because of the good screening capability of O-PSM, this method can also be used for other diseases to provide theoretical support for the development of better methods of diagnose, stratification and personalized treatment plans for patients. For example, it has recently been shown that tumor-specific immuno-profiling based on biomarkers can be used in bladder cancer patients treatable with immune checkpoints inhibitors [87]. Second, we also performed permutation on TCGA BRCA data to more accurately confirm the efficacies of these five miRNAs as BRCA clinical diagnosis markers. However, the data after random perturbation could not pass O-PSM, further validating the strict screening conditions of O-PSM. Third, we analyzed the prognostic impact of only one marker (hsa-miR-342-5p) and may have overlooked information about the prognostic impact of other markers on BRCA patients. Fourth, there are only a few studies focused on tissue and blood miRNAs in BRCA; thus, the research studies in comparison with previously reported biomarkers were limited, which proves that this research is of great significance; therefore, further study is necessary.

In the future, we hope that more scientists can further explore the miRNAs (hsa-miR-139-5p, hsa-miR-331-3p, hsa-miR-342-5p, hsa-miR-486-5p, and hsa-miR-654-3p) screened by O-PSM. Despite the limitations of our study, we believe that our results can be clinically significant in BRCA cases. Moreover, we hope to be able to extend O-PSM to more diseases and organs, to find effective and reliable biomarkers, and to contribute to advancements in the field of accurate non-invasive cancer diagnosis and prognostic stratification of cancer patients. The final goal is to be able to design tumor-specific personalized treatment plans for cancer patients in the next future.

## Figures and Tables

**Table 1:** The Area Under Curve (AUC) and Confidence Interval (CI) value of i9k60 in typing (GSE81002).

Subtype	Combined AUC	CI
Basal-like	0.840	78.94~89.15%
Normal-like	0.876	83.05~92.13%
Luminal A	0.680	62.66~73.39%
Luminal B	0.686	62.30~75.00%
HER-2 (+)	0.686	60.21~76.95%

**Table 2:** The AUC and CI value of i9k60 in Staging (GSE97811).

Stage	Combined AUC	CI
1	0.657	51.78~79.70%
2	0.723	59.08~85.48%
3	0.615	62.12~85.03%

## Data Availability

Publicly available datasets were analyzed in this study. The data can be found at https://xenabrowser.net/ and https://www.ncbi.nlm.nih.gov/geo/ (accessed on 28 August 2022). The R package is available at https://github.com/SuFei-lab/OPSM.git (accessed on 28 August 2022).

## Supplementary Materials

The following supporting information can be downloaded at: https://www.mdpi.com/article/10.3390/genes13111931/s1, Figures S1–S3: Supplementary Figures information. Figure S1, the single ROC of each miRNA and their combined ROC in test set of i9k60. Figure S2, the single ROC of each miRNA and their combined ROC of i9k60 in tissue independent test set 1. Figure S3, the single ROC of each miRNA and their combined ROC in tissue independent set 2of i9k60; Table S1: indicators of primary synergy miRNAs in 12 evaluate sets. Table S2: The AUC and ci value of i9k60 in evaluate set. Table S3: The AUC and ci value of i9k60 in test set. Table S4: The AUC and ci value of i9k60 in tissue independent set 1. Table S5: The AUC and ci value of i9k60 in tissue independent set 2. Table S6: The AUC and ci value of i9k60 in blood independent set. Table S7: Comparison of Methods.
