# Supplementary material for: Integrated Tissue and Blood miRNA Expression Profiles Identify Novel Biomarkers for Accurate Non-Invasive Diagnosis of Breast Cancer: Preliminary Results and Future Clinical Implications
Source: Genes (Basel). 2022 Oct 24;13(11):1931. doi: 10.3390/genes13111931 (PMC9690091; doi:10.3390/genes13111931)
Supplement: Supplementary file 1 [file genes-13-01931-s001.zip › Supplementary Figures information.pdf]

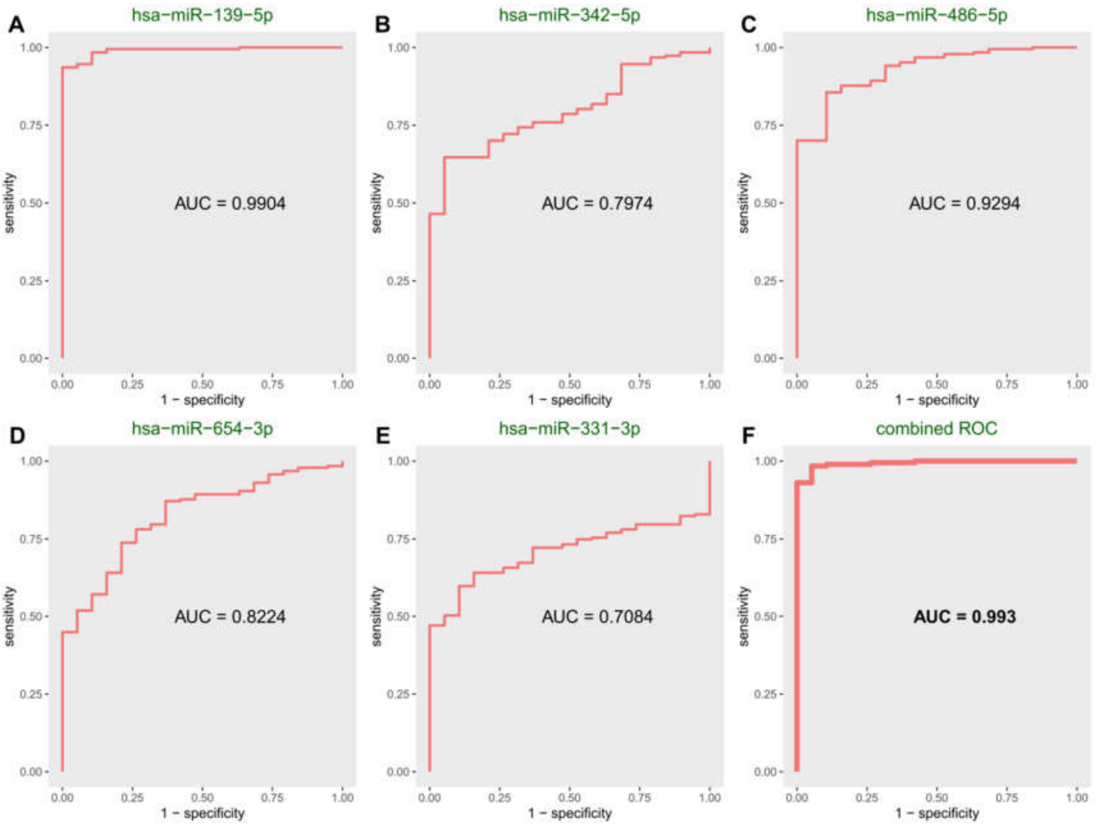


**Supplementary Figure S1.** The single ROC of each miRNA and their combined ROC in test set of i9k60: (A-E) single ROC of each miRNA in test set of i9k60; (F) the combined ROC of the 5 miRNAs in test set of i9k60.

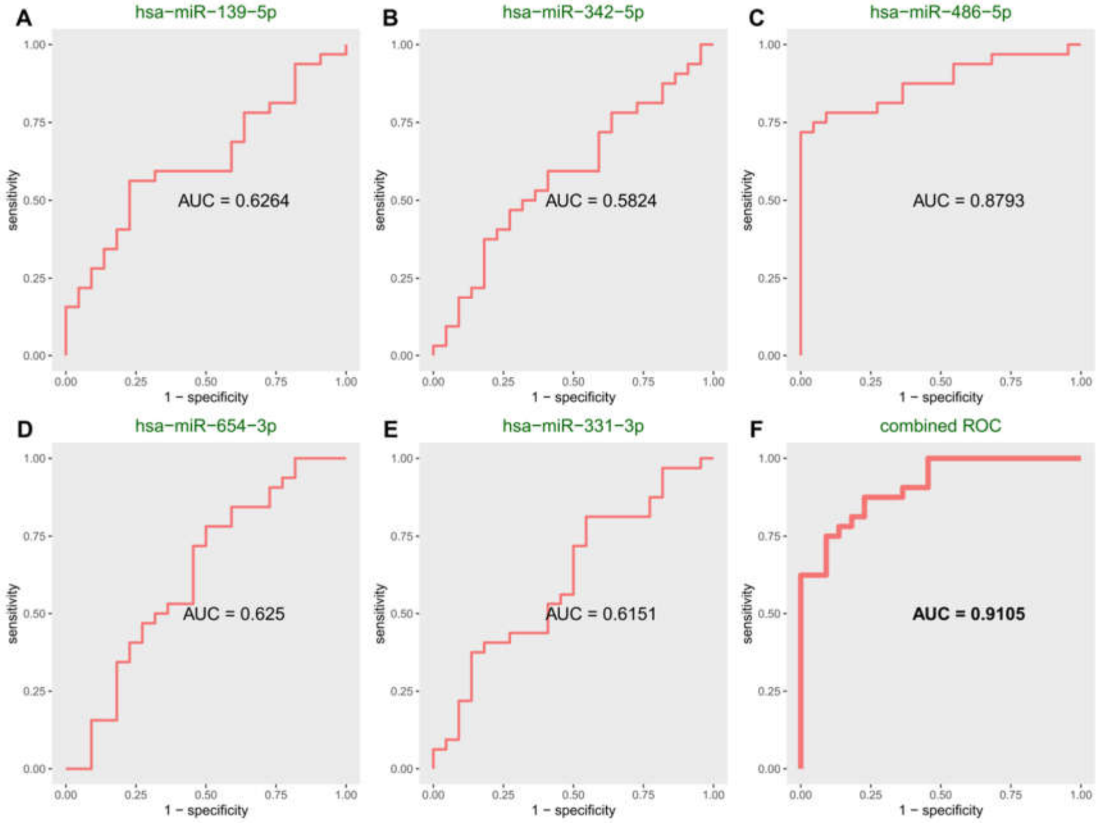


**Supplementary Figure S2.** The single ROC of each miRNA and their combined ROC of i9k60 in tissue independent test set 1: (A-E) single ROC of each miRNA of i9k60 in tissue independent test set 1; (F) the combined ROC of the 5 miRNAs of i9k60 in tissue independent test set 1.

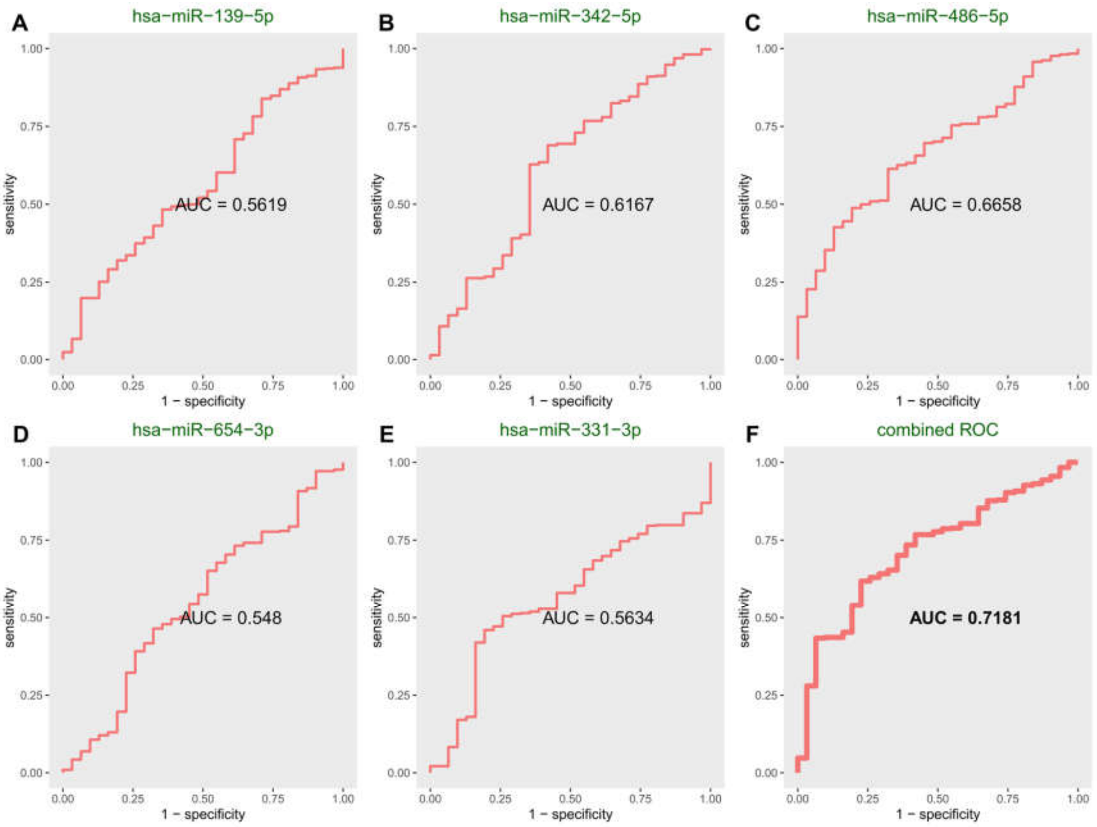


**Supplementary Figure S3.** The single ROC of each miRNA and their combined ROC in tissue independent set 2of i9k60: (A-E) single ROC of each miRNA in tissue independent set 2 of i9k60; (F) the combined ROC of the 5 miRNAs in tissue independent set 2 of i9k60.
